# Expression of Diverse Angiogenesis Factor in Different Stages of the 4T1 Tumor as a Mouse Model of Triple-Negative Breast Cancer

**DOI:** 10.34172/apb.2020.039

**Published:** 2020-02-18

**Authors:** Saba Malekian, Marveh Rahmati, Soyar Sari, Monireh Kazemimanesh, Raheleh Kheirbakhsh, Ahad Muhammadnejad, Saeid Amanpour

**Affiliations:** ^1^Cancer Biology Research Center, Tehran University of Medical Sciences, Tehran, Iran.; ^2^Department of Molecular and Cellular Sciences, Faculty of Advanced Science and Technology, Tehran Medical Sciences, Islamic Azad University, Tehran, Iran.; ^3^Department of Molecular Virology, Pasteur Institute of Iran, Tehran, Iran.

**Keywords:** Angiogenesis factor, bFGF, CD31, Triple-negative breast cancer, VEGF, VEGFR1, VEGFR2

## Abstract

***Purpose:*** Triple-negative breast cancer (TNBC) is specified by high vascularity and repetitious metastasis. Although several studies have indicated that angiogenesis has an important role in invasive breast cancer, a suitable model of TNBC that can show the exact onset of angiogenesis factors still needs to be developed. The purpose of this study is to determine the expression level of angiogenesis factors in different clinical stages of the 4T1 tumor as TNBC mouse model.

***Methods:*** Twenty mice were injected by the 4T1 cell line, and four mice selected as healthy controls. Following by tumor induction, the mice were randomly put into four groups, each contains four mice. Once the tumor volume reached to the early stage (<100 mm^3^), intermediate stage (100-300 mm^3^), advanced stage (300-500 mm^3^), and end stage (>500 mm^3^), they were removed by surgery. Then, the expression levels of Hif1α, VEGFR1, and VEGFR2 genes, as well as tumor markers of VEGF, bFGF and CD31, were evaluated by qPCR and immunohistochemistry (IHC) respectively. The statistical analysis was done by SPSS version 16.

***Results:*** TNBC tumors were confirmed and multi-foci metastasis in the lung were seen. The mRNA and protein expression levels of the angiogenesis factors increased in the early stage and as the tumor grew, their expression level enhanced dramatically.

***Conclusion:*** The 4T1 syngeneic mouse tumor may serve as an appropriate TNBC model for further investigation of the angiogenesis and therapies. Moreover, angiogenesis factors are induced before the advanced stage, and anti-angiogenesis therapy is necessary to be considered at the first line of treatment in TBNC.

## Introduction

Triple-negative breast cancer (TNBC) is an invading type of heterogeneous phenotype breast cancer, which is diagnosed by the lack of progesterone receptor (PR), estrogen receptor (ER), and human epidermal growth factor receptor 2 (HER2), and reports for ~15% of all breast carcinomas.^1^ TNBC is more difficult to treat than the other types of breast cancer because hormone therapy is more common to target one of the above mentioned receptors. Although the central role of angiogenesis in cancer progression has been established previously, it is still unclear whether anti-angiogenesis progressed in TNBC model.^2^ Generation of an ideal animal model of the tumor microenvironment that most mimic human tumor is challengeable. Currently several mice models, such as genetically engineered mouse models and patient-derived tumor xenograft, with some strengths, have been introduced, but their development is time-consuming and expensive.^3-5^ The 4T1 is an immunocompetent syngeneic mouse tumor that is known as TNBC model.^3,6,7^ The tumor growth and metastatic spread of 4T1 tumors also resemble human breast cancer.^6^ The role of angiogenesis in TNBC is more outstanding than other breast cancer types, because the lymphatic vessel density, microvessel density (MVD) and vascular invasion which are related to vascular endothelial growth factor (VEGF) family are momentous in TNBC.^8-10^ VEGF as a signal protein stimulates the formation of new blood vessels during embryonic development (vasculogenesis) and angiogenesis.^11^ Overexpression of VEGF during cancers leads to the expansion of tumor.^12^ VEGF as the most substantial angiogenesis factor in breast cancers,^13^ promotes the early tumor formation accordingly, effects on subsequent tumor development.^14^ For instance, VEGF expression is higher in sera of stage III breast cancer patients as compared to stage I or II and healthy subjects.^15,16^ Moreover, VEGF receptor signaling is associated to the invasive and metastatic activities and VEGFR receptor 1, 2 (VEGFR1,2) are more highly expressed (VEGFR2 is more specific) in TNBC than the other breast cancer subtypes.^17^ In addition of VEGF family, the other angiogenesis factors such as fibroblast growth factor (FGF), transforming growth factor beta-1 (TGFβ-1), platelet-derived endothelial cell growth factor (PDGF), placenta growth factor (PGF), and pleiotrophin are expressed by invasive human breast cancers.^18^ Moreover, the major key factor to angiogenesis activation is hypoxia-inducible factor-1α (HIF1α).^19^ However, HIF1α is not an only specific marker for TNBC, it can be used with other angiogenic markers to assist the better conclusion.^20^ Since the 4T1 mouse model is known as the equivalent of TNBC, to our knowledge, there is no comprehensive research on main angiogenesis factors in different stages during 4T1 tumor growth. Thus, the aim of this study is to evaluate the exact expression of angiogenesis factors in different stages of 4T1 tumor both in mRNA and protein levels.

## Materials and Methods

### Cell line and culture conditions

The 4T1 mouse mammary tumor cell line was obtained from the Pasteur Institute (Iran, Tehran). Cells were cultured in Dulbecco’s modified Eagle’s medium (DMEM) supplemented with 10% fetal bovine serum (FBS) and 1% penicillin/streptomycin. Cells were maintained at 37°C in the presence of 5% CO2 and 95% humidity atmosphere. Cells were sub cultured in a 70-80% confluence within 2-3 days of post culture and were harvested by 1x trypsin-EDTA (Gibco Co., Germany).

### Animals

Female BALB/c wild-type mice were prepared from the Pasteur Institute (Iran, Tehran). All the animals recruited in this study were 8±12 weeks old. Mice were kept under routine conditions in the animal care facility (Imam Khomeini Hospital, Tehran) and were fed by commercial mouse diet food with a 12-hour light-dark cycle. Animals were carefully kept under surveillance throughout the experiment for signs of pain and severe stresses such as decreased in eating or drinking, reduced activities and weight loss.

### Mouse 4T1 tumor model

0.1ml DMEM containing 10^6^ 4T1 cells/μL was injected into the right flank of each mouse. Bodyweight and tumor volume of all animals were measured every other day from beginning until the end of the study. Tumor volume of each mice was quantified using a digital caliper with the bellow equation.

$$Tumor\;volume\;=\frac{Lenght\;\times Width^{2}}{2}$$

The sample size of each group is also computed according to the following formula^21^ in which “E” is the degree of freedom of analysis of variance (ANOVA).

E = Total number of animals − Total number of groups.

After tumor induction, the animals were randomly grouped into four groups, each contains four mice. Following by tumor volume reached to the following sizes; 60-100 mm^3^ (early stage), 100-300 mm^3^ (intermediate stage), 300-500 mm^3^ (advanced stage), 500-1000 mm^3^ (end-stage), they were removed by surgery and divided into two parts. One of them kept into RNA latter for later molecular analysis and the second part preserved in formalin for further pathological analysis in order to keep safe without any changing.

### RNA extraction

Total RNA isolated from tissue samples using RNAX-Plus kit (Cinna Gen, Tehran, Iran) according to the manufacturer’s recommendations. Total RNA was qualified and quantified by 1.5% gel agarose electrophoresis and NanoDrop spectrophotometer (Thermo Scientific Nanodrop 2000) respectively.

### Quantitative real-time PCR (qRT-PCR)

Samples of cDNA were synthesized from total RNA (1000 ng) using the cDNA synthesis kit (Takara Bio, Otsu, Japan). According to the manufacturer’s recommendations, 100 ng/µL of template cDNA was put to the final volume of 20 μL of the reaction mixture. qPCR was performed with the SYBR Green method (Yekta Tajhiz Co, Cat No: YT2551) by real-time PCR system (Bioneer, Exicycler™ 96). Reverse transcriptase PCR cycle parameters included 5 minutes at 95°C, then 40 cycles of denaturation (15 seconds at 95°C), annealing (40 seconds at 60°C) and elongation process (10 seconds at 72°C). GAPDH was used as the housekeeping gene. The primers are designed by Primer3, Oligo analyzer and Gene runner software and were also checked in integrated device technology. The sequences of them are in Table 1.

**Table 1:** Sequence of primers of Hif1, VEGFR1, VEGFR2 genes and GAPDH

**Gene**	**Sequence of primer**	**Length of product**	**Tm (°C)**
VEGFR1	Forward:5ʹ GCACATGACGGAAGGAAGAC 3ʹ	187	60
	Reverse:5ʹ TTCGCAGTTCAGCAGTCCTA 3ʹ		
VEGFR2	Forward:5ʹ ACGAGGAGAGAGGGTCATCT 3ʹ	93	60
	Reverse1:5ʹ GACACACTCTCCTGCTCAGT 3ʹ		
GAPDH	Forward:5ʹ TCGGAGTCAACGGATTTG 3ʹ	219	60
	Reverse:5ʹ CCTGGAAGATGGTGATGG 3ʹ		
HIF1α	Forward:5ʹ CCAACCTCAGTGTGGGTACA 3ʹ	254	60
	Reverse:5ʹ CGGCTCATAACCCATCAACT 3ʹ		

### Immunohistochemistry

The expression of VEGF, bFGF, CD31 proteins in different stages of tumors was studied by immunohistochemistry method. Formalin-fixed and paraffin-embedded of blocks of 4T1 tumors were selected. Briefly, 5 μm-thick tissue sections were deparaffinized with xylene, then gradual rehydration with grades of alcohol were done, followed by washing with PBS several times. The non-specific antibody binding sites were blocked with blocking solution. They were incubated by primary antibodies for 24 hours at 4°C and then were incubated again with specific secondary antibodies. DAB staining was also carried out for immunohistochemical detection. Nuclear staining in immunohistochemistry (IHC) panel is performed by hematoxylin staining (background staining). The different stages were confirmed by size and hematoxylin/ eosin staining (H & E). In microscopic methods, four hot spot points selected and the average of the microvessels density of CD31 and VEGF accounted. For bFGF, modified Allred scoring system is considered.

### Statistical analysis

The statistical analysis was performed using the REST 2009 and SPSS 22 softwares. All data are shown as the mean ± SD. Statistically significant differences between groups were determined by using t independent student test. *P* < 0.05 was considered statistically significant. The quantitative results of IHC were analyzed by counting positive endothelial cells.

## Results

### Tumor-bearing mice

**Figure 1:**
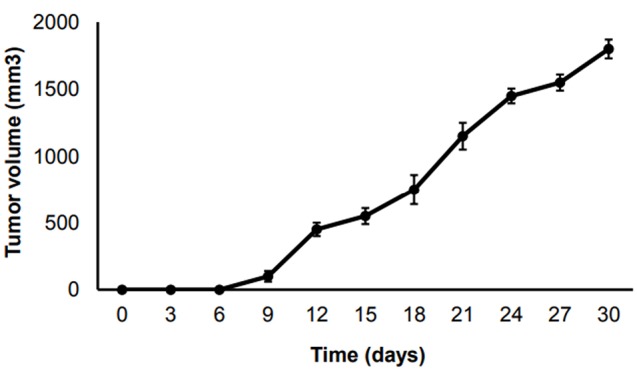
The growth curve of 4T1 tumors in TNBC mouse model in different time points.

Following by transplanting the 4T1 cells, tumors were formed in 16 mice. Then, the tumors volumes were measured and classified into four groups randomly. Three groups with four mice were used for the early, intermediate and advanced stages. Remaining four animals were used in end stage group. Tumor growth curves were consistent with other studies in our research center (Figure 1). The results showed that 6 days after the tumor induction, the tumors grew consistently until day 9, and then went up substantially in day 12 when the tumor volume reached 500 mm^3^ (advanced stage). Then the tumor growth enhanced gradually till the end stage.

### Different angiogenesis factor in TNBC tumors

Tumors of all mice in all stages of growth as compared to normal breast tissue was analyzed for angiogenesis factors such as Hif1α, VEGFR1, and VEGFR2 at the mRNA levels. The results showed that the gene expression of Hif1α, VEGFR and VEGFR2 started at the early stage when the volume of the tumor is less than 100 mm^3^, then went up dramatically in advanced and end stage as compared with healthy ones.

**Figure 2:**
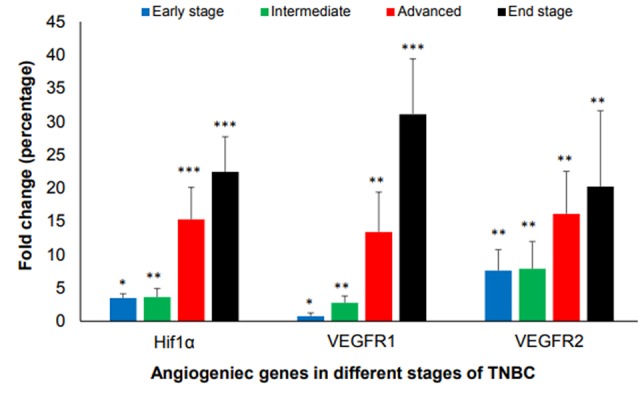
Expression of HIF1α, VEGFR1, and VEGFR2 in different stages of tumor growth in comparison to normal healthy groups. Tumor volume of <100 mm3 is in early- stage, 100-300 mm^3^ is in the intermediate stage, 300-500 mm3 is in the advanced stage and 500-1000 mm^3^ is in end-stage. (*P <  0.05, **P <  0.01, ***P <  0.001).

Quantitatively speaking, in early stages of tumor growth, the average relative expression level of Hif1α, VEGFR1 and VEGFR2 were 3.48- (*P* < 0.05), 0.75- (*P* < 0.05) and 7.6-fold (*P* < 0.01), respectively, in comparison to healthy controls. In intermediate stages of tumor growth, the average relative expression level of Hif1α, VEGFR1 and VEGFR2 were 3.6- (*P* < 0.01), 2.74- (*P* < 0.01) and 7.85- fold (*P* < 0.01), respectively. In advanced stages of tumor growth, the expression level of Hif1α, VEGFR1 and VEGFR2 were 15.28- (*P* < 0.001), 13.40- (*P* < 0.01) and 16.13 fold (*P* < 0.01). In end stages of tumor growth, the expression level of Hif1α, VEGFR1 and VEGFR2 were 22.42- (*P* < 0.001), 31.06- (*P* < 0.001) and 20.22-fold (*P* < 0.01), respectively (Figure 2).

**Figure 3:**
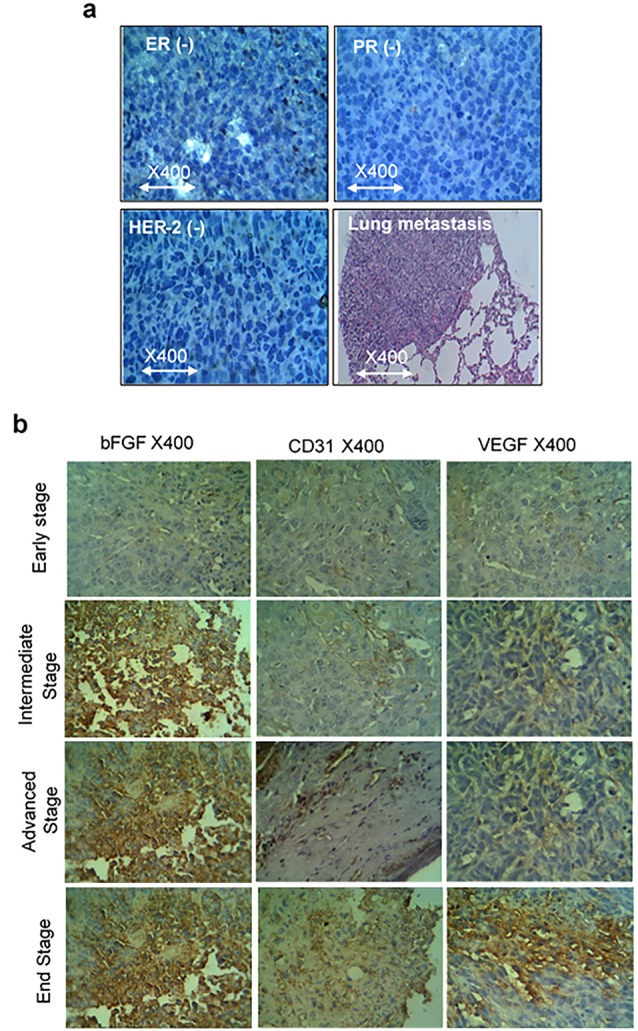
(**a**) ER, PR and HER-2 negative and lung metastasis of the 4T1 tumor. (**b**) Immunohistochemistry of bFGF, CD31, and VEGF in different stages of tumor growth in BALB/c TNBC syngeneic mice. Tumor volume of <100 mm^3^ is in early stage, 100-300 mm^3^ is in the intermediate stage, 300-500 mm^3^ is in the advanced stage and 500-1000 mm^3^ is in the end-stage.

To evaluate the validity of the triple-negative of the tumors, three markers of ER, PR and Her-2 and lung metastasis were analyzed and the results showed that 4T1 is negative for all three mentioned markers and multi-foci metastasis in the lung were seen (Figure 3a).

**Figure 4:**
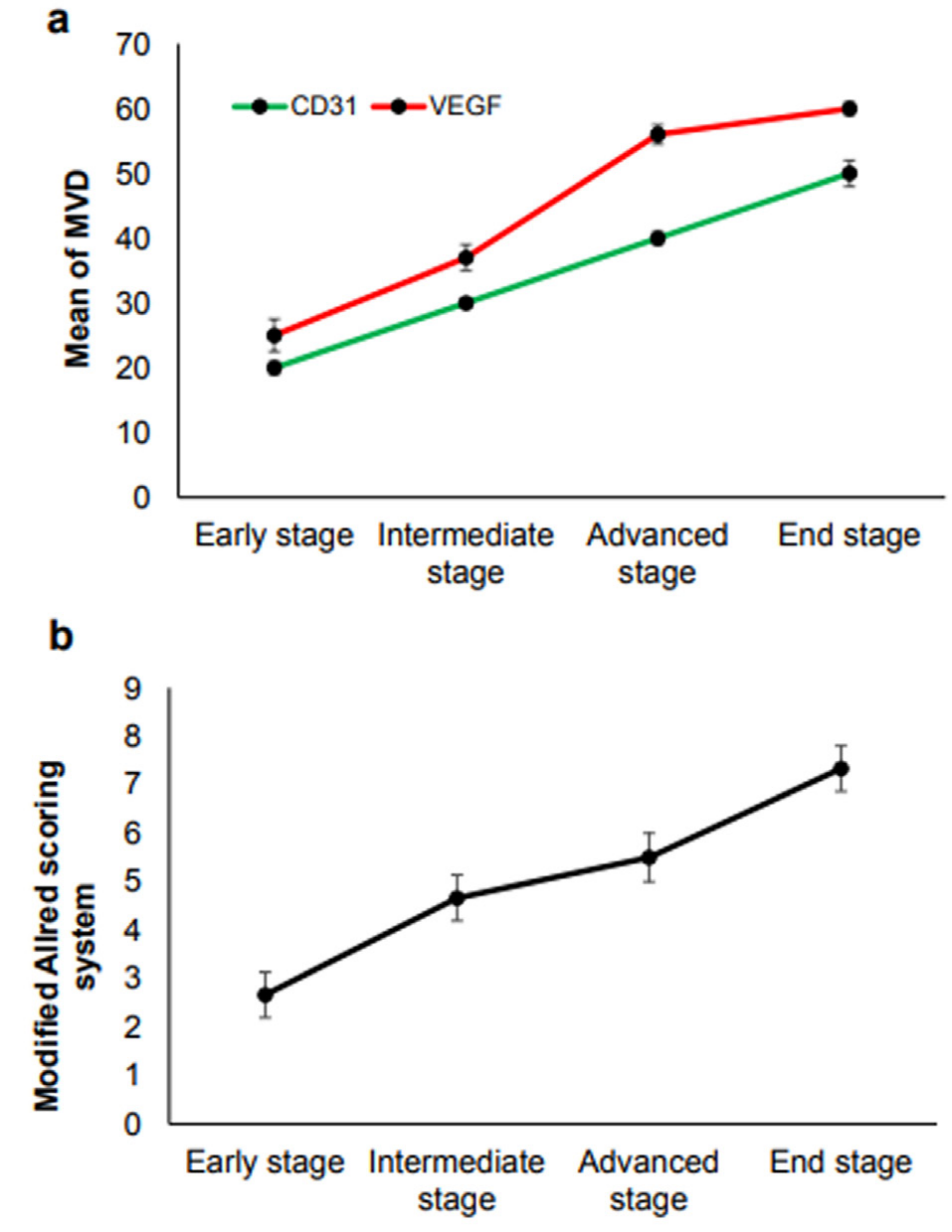
(**a**) The CD31 and VEGF (Mean of MVD),**(b)** the quantitative expression level of proteins bFGF (scoring system) in different stages of tumor growth in BALB/c TNBC syngeneic mice according to counting the staining positive endothelial cells (*P* < 0.05).

To evaluate the protein levels of angiogenesis factors in tumor tissue, the CD31, VEGF, and bFGF expressions, were analyzed by IHC (Figure 3b). Similar to gene expression results, we observed that CD31, VEGF and bFGF protein levels expression started at the early stage when the volume of the tumor is less than 100 mm.^3^ The results of mean MVD of CD31 showed that its expression is raised in a consistently form in compare to VEGF which is increased in the early stage and climbed sharply in the advanced stage and then went up moderately. The scoring system for bFGF is in agreement with the other factors and showed the enhancement of expression by the tumor growth. As shown in Figure 3b and 4, there is a correlation between the level of CD31, VEGF and bFGF protein levels in tumor tissues with pathological characteristics such as tumor stages and tumor sizes.

## Discussion

This study was undertaken to characterize some factors and markers of angiogenesis in different stages of tumor growth in a famous TNBC model in mice. Various breast cancer models in mice have been developed previously, but each model has its own strength and limitations.^18,22,23^ Even though the syngeneic models have some weakness points such as quick progression and metastasis, still, are the simplest models for research of tumor growth and metastasis.^7,24,25^ Although we have been working on the production of this model for many years successfully, we have never fully comprehended this tumor in terms of these significant angiogenesis markers. To address the main important angiogenesis factors in different stages of tumor growth, the tumor volume of each group is calculated separately. The results showed the 4T1 tumor express the most known angiogenesis factors in oncology as well. These factors belong to several different growth factor families; some belong to the PDGF supergene family such as VEGF and its receptors,^26^ others are pluripotent growth factors such as bFGF.^27^ In this study, the factors which are most highly expressed at the RNA level (HIF1α, VEGFR1, and VEGFR2) and protein level (CD31, VEGF, and bFGF) were measured. HIf1α is a common factor in the development of different tumors and breast cancers. In the experiments with TNBC patients, the results showed that HIF1α in TNBC patients are more expressed than non-TNBC and there was a considerable correlation between Hif1α expression, tumor size and pathologic stages.^28^ In syngeneic mouse models, HIF1α also promotes the growth, invasion, and metastasis of TNBC cells.^29^ In our experiment, the expression of HIF1α is initiated in the early stage when the tumor volume is less than 100 mm^3^ and as the tumor grew, the expression of HIF1 is increased (*P* < 0.05) and reached to the highest level in the end stage. Therefore it could be a significant marker in the evaluation of the tumor growth and considered in therapeutical decisions.

VEGF causes vascular permeability enhancement, as well as angiogenesis promotion, and maybe one of the most important mediators of tumor angiogenesis.^26^ As the stage of the tumor is progressed, the level of VEGF is increased. Previous studies have shown, VEGF level in serum of stage III breast cancer patients was higher than in the stage I or II breast cancer and healthy people.^15,16^ Our results is in agreement with the previous studies and indicate the mean MVD of VEGF is increased in the early stage and risen steadily in the intermediate stage, soared significantly in the advanced stage and then reached to the end stage with a gradually rise. This means that VEGF considerably enhanced tumor growth. VEGF receptor signaling is related to invasive and metastatic processes and VEGFR receptor 2 (VEGFR2) is more highly expressed in TNBC than in other breast cancer subtypes.^17^ In this study, the VEGF expression in protein level and its receptors, VEGFR1 and VEGFR2 expression in mRNA level are determined and showed the increased level of them in TNBC tumors in comparison to non-TNBC tumors. As the tumor volume is increased, the level of angiogenesis factor is increasing (*P* < 0.05). The other angiogenesis factor such as CD31 and bFGF are determined by IHC and the results showed all the angiogenesis factor is expressed in very early stage and is increased in higher stages. There was a good correlation in our assays between the mRNA and protein levels of multiple angiogenesis factors, suggesting that angiogenesis is initiated in very early stages of TNBC.

## Conclusion

The syngeneic mice model of TNBC is suitable for angiogenesis and tumor growth study. The expression of different angiogenesis factors at both mRNA and protein levels is started at early stage and increased at intermediated stages of TNBC. Given these results and that angiogenesis is independent of ER status, anti-angiogenesis therapies should be considered as the first line of treatment in TNBC. Further studies should be done for better understanding angiogenesis using mice 4T1 as a TNBC model.

## Ethical Issues

The project was approved by the guidelines of the Ethics Committee of Tehran University of Medical Sciences, Iran (IR.TUMS.IKHC.REC.1397.044).

## Conflict of Interest

Authors declare no conflict of interest in this study.

## Acknowledgments

This project has been conducted by a grant from Cancer Research Center of Cancer Institute of Iran (Sohrabi Cancer Charity, grant No: 37775-202-01-97) and Cancer Biology Research Center (grant No 97-01-180-37875).
